# Polish Scientists in Fish Immunology: A Short History

**DOI:** 10.3390/biology4040735

**Published:** 2015-11-11

**Authors:** Willem B. Van Muiswinkel, Andrzej Pilarczyk

**Affiliations:** 1 Department of Animal Sciences, Cell Biology and Immunology Group, Wageningen University, P.O. Box 338, 6700 AH Wageningen, The Netherlands; 2 Institute of Ichthyobiology and Aquaculture of PAS (Polish Academy of Sciences), Gołysz, 43-520 Chybie, Poland; E-Mail: andrzej.pilarczyk@golysz.pan.pl

**Keywords:** Poland, history, biography, fish immunology, fish health, vaccination, aquaculture

## Abstract

This review describes the role played by Polish scientists in the field of fish immunology and vaccination starting around 1900. In the early days, most publications were dealing with a description of relevant cells and organs in fish. Functional studies (phagocytosis, antibody response) came later starting in the late 1930s. Detailed papers on fish vaccination were published from 1970 onwards. Another important development was the unraveling of neuro-endocrine-immune interactions in the 1970s until today. Around 1980, it became more and more clear how important immunomodulation (stimulation or suppression by environmental factors, food components, drugs) was for fish health. The most recent findings are focusing on the discovery of genetic factors, signaling molecules, and receptors, which play a crucial role in the immune response. It can be concluded, that Polish scientists made considerable contributions to our present understanding of fish immunity and to applications in aquaculture worldwide.

## 1. Introduction

According to Silverstein the start of “modern” immunology was in the early 1960s [1]. These were the days that classical immunochemistry gave way to modern immunobiology. “Modern” fish immunology took off slightly later and was regarded usually as a subdivision of comparative immunology or veterinairy immunology. Today, fish immunology has developed into a flourishing and independent scientific field with tight links with general immunology and aquaculture. A concise history of fish immunology was published earlier by Van Muiswinkel [2] and Van Muiswinkel & Nakao [3]. In the present review we are describing in more detail the role played by Polish scientists in the development of our present knowledge of fish immunology and vaccination. It is not the intent of the authors to evaluate the scientific merit of the work discussed, but to provide the reader with a short historic overview and to give some biographic information on the people behind these studies. Publications from before 1950 or in languages other than English (e.g., Polish) are sometimes not found by today’s database searches on the internet. Fortunately, the modern libraries of many universities/institutes are so well organized, that old publications can be found. 

Aquaculture has always been an integral part of traditional farming in Poland, where the harvest from fish ponds provided the farmer with a “second crop”. Already in 1573 the pond master, Olbrycht Strumieński, published a book entitled “*About managing, pond building, and stocking. Also about digging*, *water leveling, and conducting. A book useful for every farmer*” [4]. Over the centuries, Polish farmers have developed a lot of practical experience with fish and their diseases. This may explain the interest for fish immunology and fish health in scientists with a Polish background.

Taking into account, that this publication is describing a period of more than 100 years of science and that scientific progress today is amazingly fast, it implies that this historic review offers only a limited picture of the past.

## 2. Histology and Hematology

**Figure 1:**
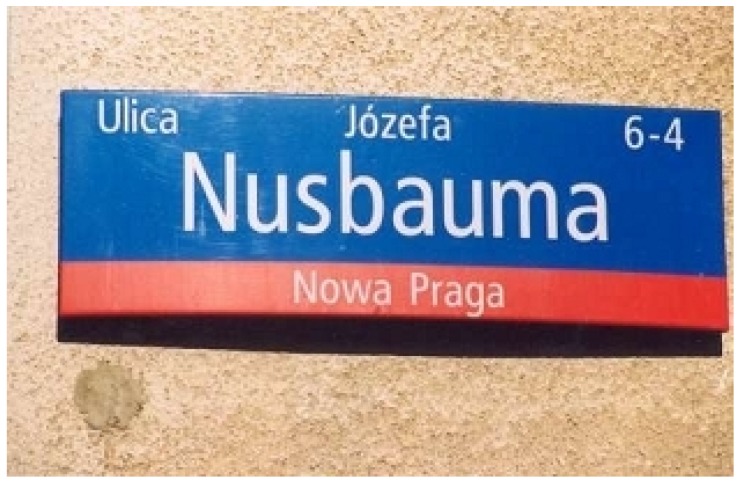
A street in Warsaw (Poland) is named after the famous Darwinist and comparative anatomist Józef Nusbaum (1859–1917) from the University of Lwów (today Ukraine).

There have been early histological studies by Nusbaum (Table 1, Figure 1) describing the development and morphology of the thymus in trout and goldfish [5]. He was convinced, that thymocytes developed directly from epithelial cells. In the days around 1900, the role of hemopoietic precursor cells was not yet known. Moreover, the function of the thymus as primary lymphoid organ and source of regulatory and cytotoxic T cells was unknown as well.

**Figure 2:**
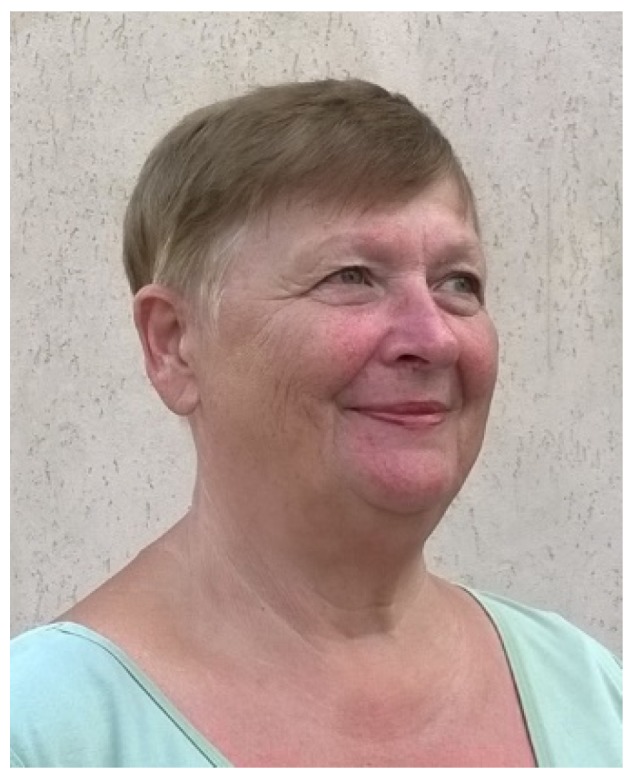
Antonina Sopińska joined the Institute of Biological Bases of Animal Diseases, Subdepartment of Fish Diseases and Biology, University of Life Sciences, Lublin (Poland) in 1973. She received her DSc (“Habilitation”) degree based upon a thesis entitled “The effect of immunostimulation on cellular defense under different conditions in carp” in 1991. Subsequently she was appointed as professor and head of the subdepartment. Her laboratory has performed many studies on the immune mechanisms of fish and the impact of various external factors (e.g., season, stress, pollution) on the immune system of fish. The picture from 2015 was obtained from A. Sopińska and is used with her permission.

In the 1980s it was shown by Sopińska (Table 1, Figure 2), that relatively simple hematological techniques, such as differential blood cell counts, can provide us with interesting data about important cell types, e.g., lymphocytes and granulocytes. Amazingly enough, the total number of blood leucocytes of carp between 1 and 28 months of age did not differ significantly [6]. However, the percentage of granulocytes is increasing with age. In the same study it was shown, that male carp during spawning show lower lymphocyte, but higher neutrophil counts than females. A possible explanation for these differences between the sexes is a relative high stress response in male carp during the spawning season (May–June). This idea is supported by another study from Sopińska in 1984 [7]. This publication makes clear, that transportation stress causes a decrease of lymphocytes, but an increase of neutrophils in carp blood. Today it is known that even mild stress can cause a redistribution of important cell types between organs like blood and head kidney. Engelsma *et al*. [8] showed, that temperature stress in carp reduces the number of circulating B-lymphocytes. However, the percentage of granulocytes nearly doubled in blood, but decreased in the head kidney. These changes in leucocyte populations were accompanied by lower antibody titers after immunization. In other words, stress may cause a shift from acquired immunity (B-lymphocytes ↓) to innate immunity (granulocytes ↑).

**Table 1:** Polish scientists in the field of fish immunology starting their career between 1900 and 1985 ^a^.

Scientist (Figure)	Subject	Reference (Year)
J. Nusbaum (Figure 1)	Thymus development	[5] (1901)
J. Borowik (Figure 3)	Phagocytosis	[9] (1922)
S.F. Snieszko (Figure 6A)	Remark on vaccination (*Aeromonas punctata*)	[18] (1938)
	Oral immunization	[19] (1949)
	Fish vaccination	[20] (1970)
	Stress and diseases	[36] (1974)
F. Pliszka (Figure 4)	Specific immune response and phagocytosis	[10] (1939)
		[11] (1939)
M.M. Sigel ^b^	Secondary response in sharks	[15] (1966)
	Immunoglobulin structure in sharks	[14] (1967)
B. Plytycz (Figure 10)	Ontogeny of neuro-endocrine organs	[38] (1974)
	Morphine modulation of inflammation	[39] (1996)
		[40] (1997)
	Effect of stress on inflammation	[55] (1995)
		[41] (1999)
A. Pilarczyk (Figure 5)	Immune response against *Yersinia ruckeri*	[17] (1982)
	Disease resistance in carp lines	[47] (1995)
	Genetic differences in disease resistance	[51] (2009)
A. Sopińska (Figure 2)	Effects of season, stress	[6] (1983)
		[7] (1984)
	Vaccination and immunomodulation	[22] (1996)
		[29] (2011)

^a^ The first publication is regarded as the career starting point in fish immunology; ^b^ M. Michael Sigel (1920–1995) emigrated from Poland to the USA in 1937. For additional information on M.M. Sigel see Van Muiswinkel [2].

## 3. Innate and Acquired Immunity

**Figure 3:**
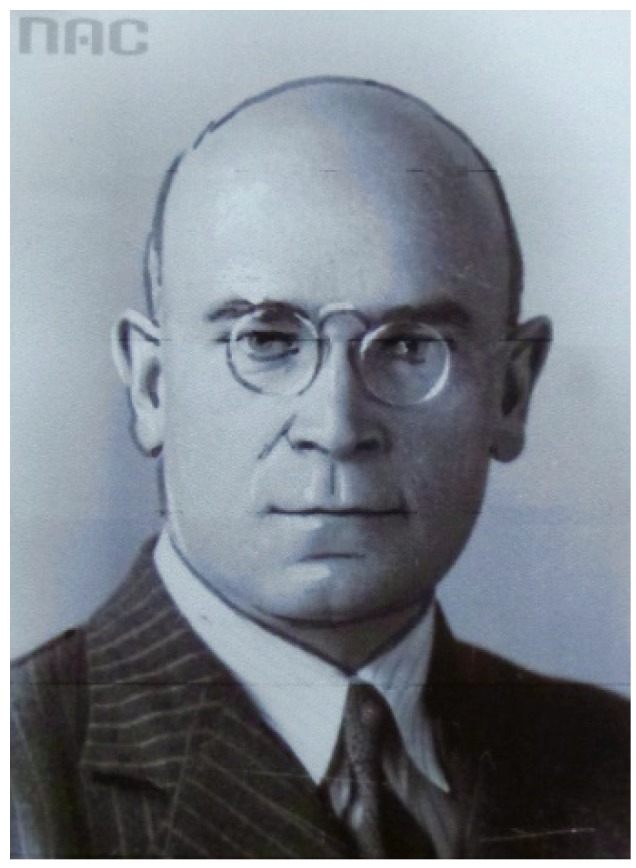
Józef Borowik (1891–1968) was an ichthyologist, who published a book on Fish Physiology in 1922 [9]. Here it is mentioned that fish leukocytes can “eat” bacteria. Borowik became a successful director of the Baltic Institute at Gdańsk (Poland) during the years 1927–1950 (with a break during World War II). His picture was obtained from Narodowe Archiwum Cyfrowe (NAC, National Digital Archives), Warsaw (Poland) and is used with permission.

The ichthyologist and organizer, Borowik (Table 1, Figure 3), mentions in his book on “Fish Physiology”, that fish leucocytes are mobile cells, that can “eat” bacteria [9]. In other words: he describes already in 1922 this form of innate immunity, which is important for the defense against diseases.

**Figure 4:**
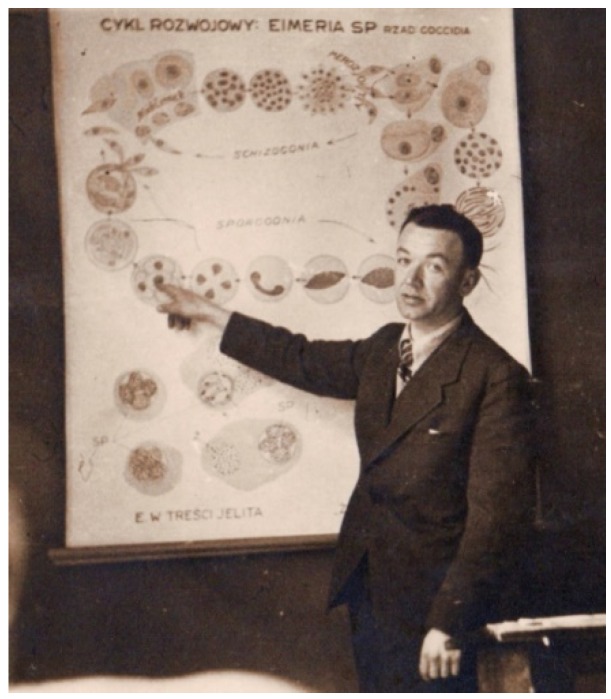
Franciszek Pliszka (1909–1956) teaching fish diseases in 1943. He received his Ph.D. degree based upon a thesis entitled “Obserwacje nad oddychaniem ryb (Observations on the respiration of fish)” from the Faculty of Veterinary Medicine, Agricultural University of Warsaw (SGGW, Poland) in 1937. In the period between 1938 and 1942 he was appointed to the Department of Ichthyology and Fisheries of the Jagiellonian University, Kraków (Poland) and the Fisheries Biology Station at Mydlniki (Poland). At that time he started with his work on bacterial and parasitic diseases of fish. In 1939, he was one of the first people in the world to show that fish were able to mount specific immune responses [10,11]. In the years 1942–1944 he was head of the Laboratory of Fish Diseases, Veterinary Institute at Pulawy (Poland) belonging to the SGGW. He took part in the Warsaw Uprising in 1944. In 1945 he received his DSc degree (“Habilitation”) from the SGGW. In 1952 followed his appointment as professor in Fish Biology at the Higher School of Agriculture, Olsztyn (Poland). For obituary in Polish see [12].

**Figure 5:**
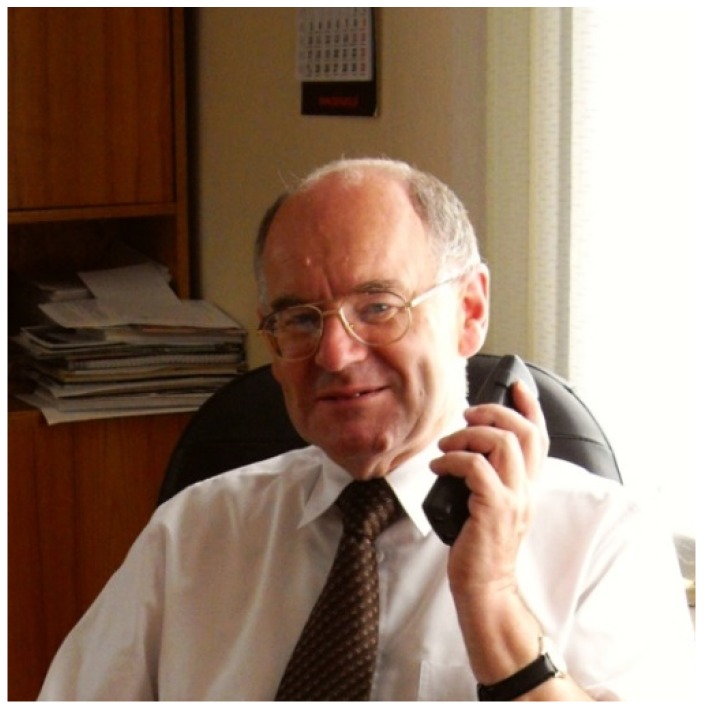
Andrzej Pilarczyk at his office in 2009. He obtained his PhD degree in Biological Sciences from the Agricultural University, Szczecin (Poland) in 1982. His DSc (“Habilitation”) degree in Fisheries based on a dissertation entitled “Impact of genetic and dietary factors on the immune response of carp” at the same university in 1998. In the period between 1972 and 2013 he has been appointed at the Institute of Ichthyobiology and Aquaculture of PAS (Polish Academy of Sciences), Chybie (Poland) as assistant, senior assistant, associate professor, and director. In 1999, he became a professor at the Department of Food Engineering, Polytechnic University of Bielsko-Biala (Poland). He has published extensively on genetic aspects of the immune response in different carp lines.

Pliszka (Table 1, Figure 4) describes in 1939 as one of the first in the world, that fish can deal with invading bacteria in two ways: phagocytosis and agglutinin production [10,11]. In his studies he used killed *Pseudomonas punctata* (today *Aeromonas hydrophila*) to immunize carp. At temperatures between 18 and 20 °C the animals were producing specific agglutinins with a peak three weeks post immunization. No response was seen at 9–11 °C. This observation illustrates the effect of temperature on the immune system of fish. By using a short term culture of spleen cells he was able to observe phagocytosis of bacteria by macrophages and/or granulocytes using a powerful microscope. In later years it became clear, that these agglutinins were in fact immunoglobulin (Ig) molecules produced by lymphoid cells [13]. The structure and function of Ig molecules has been subject of study by many groups in the world during the 1960s and 1970s. One of these groups in the USA was headed by Sigel (Table 1). He showed, that elasmobranchs (e.g., sharks) produce both pentameric (19S) and monomeric (7S) Ig molecules [14]. It is worthwhile mentioning that sharks do not show clear secondary immune responses upon a second contact with the same antigen [15]. It is common knowledge now, that bony fish (e.g., carp, salmonids) develop immunological memory and show a faint to clear secondary (higher) response after repeated antigen injections [16]. It was shown by Pilarczyk (Table 1, Figure 5), that the route of antigen administration also had an effect on the magnitude of the subsequent antibody response [17]. Using different doses of O-antigen from *Yersinia ruckeri* he was able to show, that injection of carp gave higher responses than a short bath of the animals in water containing the O-antigen.

## 4. Vaccination

**Figure 6:**
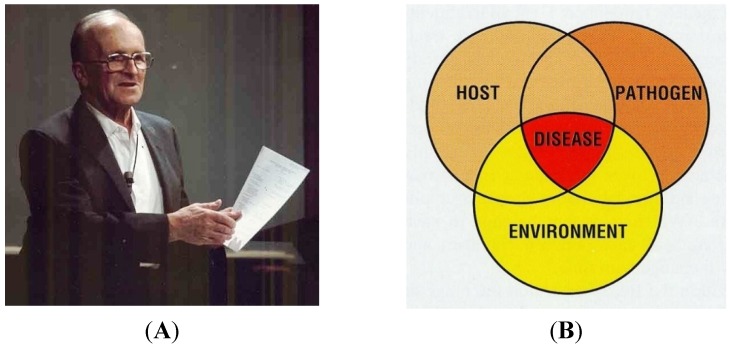
(**A**) Stanisław F. Snieszko (1902–1984) received his Ph.D. degree in Bacteriology and Chemistry from the Jagellonian University, Kraków (Poland) in 1926. In the period between 1926 and 1932, he pursued post-doctoral studies at Leipzig (Germany) and the University of Wisconsin (USA). As of 1923, he was simultaneously employed by the Department of Agricultural Bacteriology of the Jagiellonian University, where be became professor in 1937. Shortly before the war in 1939, he travelled to the USA for a conference and never turned back. During the war, he researched lobster diseases at the University of Maine, Orono (USA) and then joined the Chemical Corps at Fort Detrick, Frederick, (MD, USA), where he was involved in microbiology. In 1946, his background interest in fish led to employment by the U.S. Fish & Wildlife Service at Leetown (WV, USA) to assist James Gutsell conduct research on the use of antibiotics to treat fish diseases. He later became the 1st director of the Eastern Fish Disease Laboratory (later called National Fish Health Research Laboratory). His abilities and accomplishments in fish health are impressive, including 200 papers and several books on fish diseases, treatment, and prevention (e.g., vaccination). He retired as director in 1972. In the same year he received an Honorary Doctorate of Science from the West Virginia University, Morgantown (WV, USA). During his active life in the USA he maintained permanent contact with Polish colleagues as their problems were always close to his heart. For a comprehensive biography see [21]; (**B**) The famous “Snieszko diagram” illustrating the complex interaction between the host (genetics, immune system), pathogen (virulence), and environment (stress, climate) on development of diseases as published in 1974 [36]. This picture was taken from a booklet “What should I do? A practical guide for the fresh water farmer” with permission of the European Association of Fish Pathologists (EAFP).

In a publication from 1938 entitled “Bacteriological and serological examination of bacteria causing sepsis in carp” Snieszko (Table 1, Figure 6A) and coworkers described, that carps and rabbits were immunized against *P. punctata* after injection with killed bacteria [18]. The immune sera of these animals could be used for typing the different strains of this bacterium. Snieszko also mentions in this paper, that vaccination of fish will not be cost-effective under Polish circumstances. It took more than 10 years (after Snieszko’s move to the USA) before he reconsidered this approach again for disease prevention. In 1949 he published a comparative study on prophylaxis of furunculosis (caused by *Aeromonas salmonicida*) in brook trout by oral immunization and the antibiotic sulfamerazine [19]. Unfortunately, the drug sulfamerazine gave better protection than oral vaccination. In general, there was a tendency during the first 10–20 years after World War II to prefer chemotherapy over vaccination of fish. In a review published in 1970 Snieszko states, that vaccination by injection is more effective than via the oral route [20]. He also made the important remark that the success of a vaccine is depending on several factors, e.g., the nature of the host, the nature of the pathogen, and the conditions of the environment (see also Figure 6B and paragraph 6. Neuro-Endocrine Interaction and Stress). Interesting results from experiments by Sopińska (Figure 2), Guz, and Grawinski show, that spraying fish for a short time with a diluted vaccine is a promising option [22]. Trout could be protected with success by spraying a vaccine against enteric red mouth disease (ERM, caused by *Y. ruckeri*). In the period between 3 and 28 days post vaccination, the lymphocytes from the head kidney could be isolated and cultured in the presence of proteins derived from *Y. ruckeri*. During the whole observation period, the transformation of lymphocytes into lymphoblasts was clearly higher in cultures from vaccinated animals compared to non-vaccinated controls. Amazingly enough, no specific antibodies were found in the period after vaccination. In other words: protection can sometimes be achieved through cellular immunity in the absence of specific antibodies.

**Figure 7:**
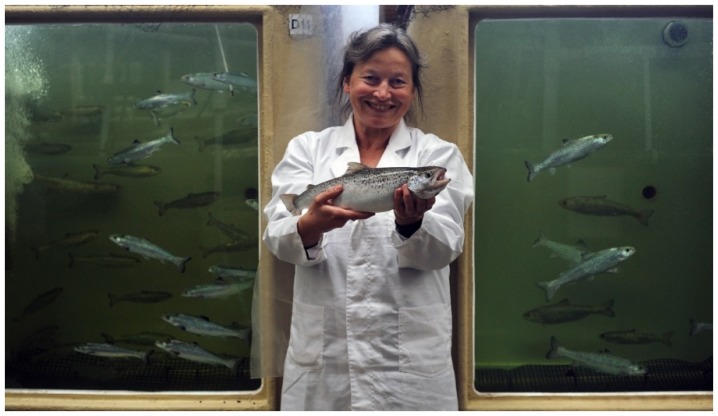
Barbara F. Nowak received her PhD degree in Aquatic Toxicology from the University of Sydney (Australia) in 1991 and her DSc (“Habilitation”) from the University of Agriculture, Szczecin (Poland) in 2004. At present she is professor in Aquatic Animal Health at the Institute of Marine and Antarctic Studies, University of Tasmania, Launceston (Australia). Her group is focusing on health of farmed fish in particular interactions between host, pathogen and environment. She is the (co)author of an impressive number of publications on Amoebic Gill Disease (AGD) in salmon, tuna health, fish immunology and histopathology.

An exciting new approach for testing vaccines was developed by the group of Nowak (Table 2, Figure 7). They first showed in a classic challenge with *Y. ruckeri*, that Atlantic salmon survived much better after immersion (bath) vaccination compared to unvaccinated controls [24]. Subsequently, they investigated the host gene expression in gills of vaccinated and unvaccinated animals after challenge. Four differentially regulated genes were found to be associated with protection following vaccination and challenge. Two of these genes were undoubtedly related to immune functioning (Ig heavy chain and selenoprotein). This approach may allow us to predict the efficacy of vaccination before challenge and therefore obviate the need for challenge in the future.

**Table 2:** Polish scientists in the field of fish immunology starting their career between 1985 and 2005 ^a^.

Scientist (Figure)	Subject	References (Year)
A.K. Siwicki (Figure 8)	Immunomodulation by drugs	[31] (1989)
	Immunomodulation by pesticides	[32] (1993)
	Immunostimulation	[25] (1994)
		[58] (1998)
		[26] (2009)
		[27] (2013)
	Immunotoxicology	[60] (1998)
I. Irnazarow (Figure 12)	Genetics of carp lines	[50] (1995)
	Polymorphism of histocompatibility genes	[51] (2009)
	Polymorphism of transferrins	[53] (2009)
	Resistance to Cyprinid herpesvirus	[52] (2009)
	Stress, genetics and disease resistance	[54] (2015)
M.K. Chadzinska (Figure 11)	Morphine modulation of inflammation	[39] (1996)
		[40] (1997)
		[41] (1999)
	Opioid receptors, stress and immune response	[42] (2009)
	CXCL8 chemokines	[43] (2010)
		[66] (2014)
	Neuroendocrine-immune interactions	[44] (2009)
	Stress, genetics and disease resistance	[54] (2015)
B.F. Nowak (Figure 7)	Cortisol effects	[45] (1999)
	Immunostimulation	[28] (2005)
	Immunoregulation and vaccination	[24] (2012)
L.R. Iwanowicz (Figure 9)	Immunomodulation during whirling disease	[35] (2004)
	PCB and decreased disease resistance	[33] (2009)
	Biomarkers for contaminant effects	[34] (2012)
		
		

^a^ The first publication is regarded as the career starting point in fish immunology.

## 5. Immunomodulation

**Figure 8:**
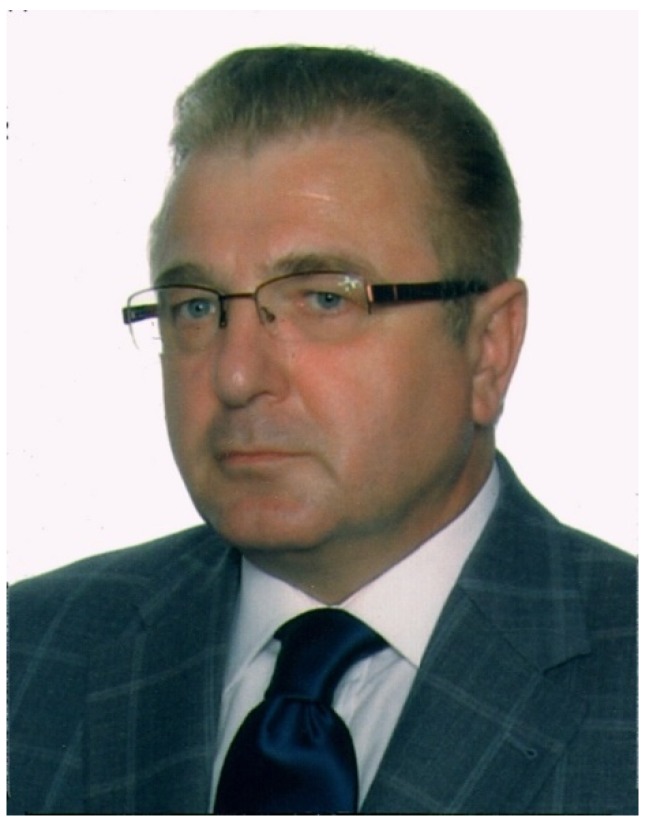
Andrzej K. Siwicki received his Ph.D. degree from the National Inland Fisheries Institute of Olsztyn (Poland) in 1983. Subsequently the DSc (“Habilitation”) degree was obtained from the Faculty of Veterinary Medicine, University in Olsztyn (Poland) based upon his thesis entitled “Modulation of cellular and humoral immunity by levamisole in fish” in 1990. In 1995, he was appointed as Professor of Veterinary Medicine at the University in Olsztyn. At present, he is head of the Department of Microbiology and Clinical Immunology, Faculty of Veterinary Medicine, University in Olsztyn as well as head of the Department of Pathology and Immunology, National Inland Fisheries Institute, Zabieniec near Warsaw, Piaseczno (Poland). He and his co-workers have published around 500 publications (> 250 refereed papers) on clinical and experimental immunology, immunotoxicology, immunomodulation (including vaccines), diagnosis, and therapy of infectious diseases in fish as well as in mammals.

**Figure 9:**
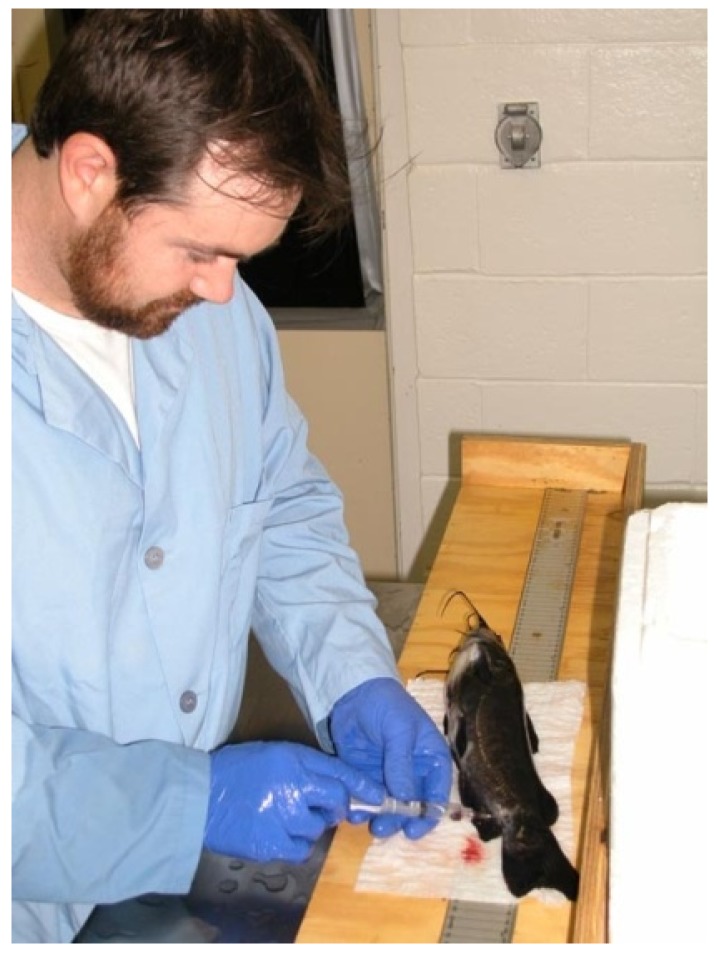
Luke R. Iwanowicz bleeding a brown bullhead (*Ameiurus nebulosus*) in his laboratory. He received his PhD degree from the University of Massachusetts, Amherst (MA, USA) in 2008 based upon a thesis entitled “Fish as Indicators of Aquatic Ecosystem Health: from the Lab to the Field”. Today he is a Research Biologist at the USGS, Leetown Science Center, Fish Health Branch, Kearneysville (WV, USA). His interests include the effects of emerging infectious diseases (viruses) and physiological disruptors (endocrine interacting chemicals) on the immune system and responses in fish. He published around 60 scientific publications. Two of these papers received the title “Paper of the Year” from the Journal of Aquatic Animal Health in 2004 and 2011.

Immunostimulants are a group of biological and synthetic compounds that enhance innate immunity in various animal groups. Immunostimulants such as β-glucans (from yeast and other sources), minerals, and vitamin combinations and other products (extracts) from plants or animals may be effective in preventing diseases in aquaculture and could be given as replacement for vaccines. In a very successful study by Siwicki (Table 2, Figure 8), Anderson, and Rumsey it was shown, that substances such as glucans, chitins (from shrimp), vitamins and betaine stimulated innate immunity markers (e.g., oxidative radical release, phagocytic index), but also resistance to furunculosis (caused by *A. salmonicida*) in trout [25]. These observations were later confirmed by Siwicki and co-workers in another fish species, pikeperch (*Sander lucioperca*). Feeding of β-glucans or yeast containing products activated phagocytes, increased lymphocyte stimulation by mitogens, lysozyme, and Ig levels in serum [26]. Moreover, reduced mortality after challenge with pathogenic *A. salmonicida* was observed [27]. Comparable results were obtained by Guz, Sopinska and Oniszczuk using a medicinal herb, *Echinacea purpurea*, as food additive in guppy (*Poecilia reticulata*) [29]. Cumulative mortalities after challenge with the fish pathogen, *Aeromonas bestiarum*, were the lowest in fish supplemented with the herb. *Echinacea* can also be used as adjuvant in combination with a vaccine. Guz *et al*. showed that zebrafish (*Danio rerio*) were better protected against columnaris disease when they received both *Echinacea* and a vaccine containing killed *Flavobacterium columnare* cells before challenge [30]. Vaccination alone gave almost no protection. Not all food additives or drugs have a positive effect on immunity in fish. Siwicki (Figure 8) *et al.* showed that oxinolinic acid, used at recommended doses for the treatment of bacterial diseases, did not cause immunosupression, but oxytetracycline reduced both nonspecific and specific immune responses in trout [31]. In addition, pesticides and other organic pollutants (polynuclear aromatic hydrocarbons, polychlorinated biphenyls: PCB, tributyltin) have all been described as pollutants in the aquatic environment with an immunosuppressive effect on fish [32]. Iwanowicz (Table 2, Figure 9) *et al.* published convincing evidence that environmental pollution can have a suppressive effect on the immune system of fish. In a laboratory study they showed that i.p. injection of Aroclor 1238 (a PCB mixture) in brown bullhead, *Ameiurus nebulosus*, decreased bactericidal activity and circulating antibodies to *Edwardsiella ictaluri* [33]. In a field study, Iwanowicz and colleagues showed that brown bullheads in a PCB contaminated river had more macrophage aggregates and a reduced bactericidal and cytotoxic cell activity compared to the same species at a relative clean site [34]. It is interesting to mention that marked seasonal effects were observed in this field study. Another factor of importance is the immodulation by pre-existing diseases on the immune capacity of a fish. Densmore *et al.* showed that rainbow trout infected with *Myxobolus cerebralis* (causative agent of whirling disease) showed lower proliferative lymphocyte responses to mitogens, lower resistance to *Y. ruckeri* challenge, but greater bactericidal activity of head kidney macrophages compared to uninfected controls [35].

## 6. Neuro-Endocrine-Immune Interaction and Stress

**Figure 10:**
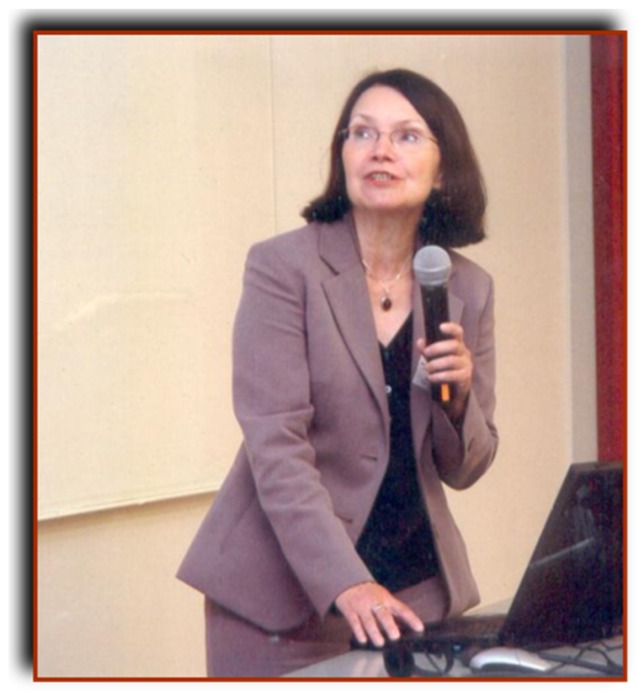
Barbara Płytycz (nee Malik) received her PhD degree on transplantation immunity in amphibia from the Jagiellonian University at Kraków (Kraków, Poland) in 1971. Her DSc degree (“Habilitation”) followed in 1981. In 1992, she became professor at the Zoology Institute of the Jagiellonian University. In the same year she founded the new Department of Evolutionary Immunobiology at the Zoology Institute. She can be regarded as an outstanding comparative immunologist. Her group is well known for their work on the evolution of the immune system with special attention to stress-immune interaction in mammals, amphibia, fish, and earthworms. Her picture was used with permission of the Jagiellonian University, (Kraków, Poland).

**Figure 11:**
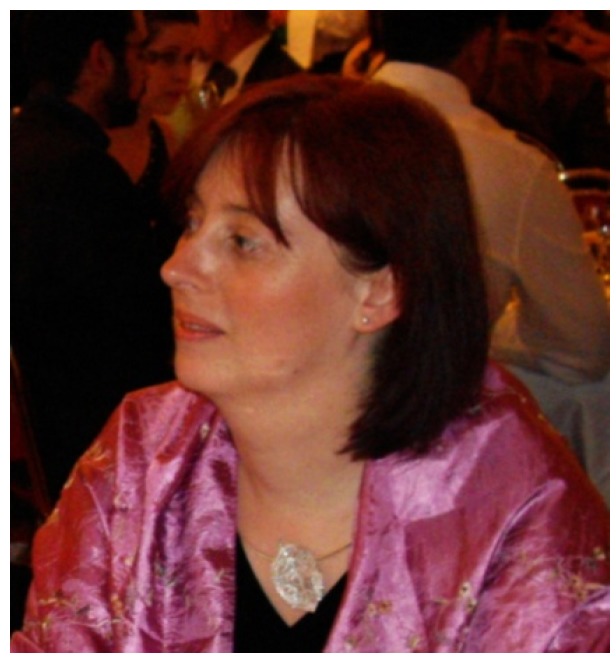
Magdalena F. Chadzińska at the 11th Congress of the International Society for Comparative & Developmental Immunology, Prague (Czech Republic) in 2009. She obtained her Ph.D. degree in Evolutionary Immunobiology from the Jagiellonian University, Kraków (Poland) in 1995. A DSc degree (“Habilitation”) for her work on “Opioid System and Innate Immunity” from the same university followed in 2009. Between 1995 and today, she went through the ranks from assistant to associate professor at the Institute of Zoology of the Jagiellonian University. Since 2011, she has been the Head of the Department of Evolutionary Immunology, Institute of Zoology. In 2012 she became Council member of the European Society for Comparative Endocrinology. She has published excellent papers on neuro-immune modulation, innate immunity, inflammation, and the biology of macrophages and neutrophils in amphibia, fish (Atlantic salmon, carp, goldfish), and mammals.

Outbreaks of disease can result from the introduction of pathogens, from malnutrition, from environmental changes, from the genetics of the fish, but usually from the interrelationship of all these factors. This idea was originally visualized by Snieszko as a set of three circles (Figure 6B) [36]. If the circles intersect enough, the conditions are favorable for an outbreak of disease. Under certain aquaculture practices (e.g., handling, transport, impaired water quality) fish can be exposed to stress. These circumstances will evoke a stress response in fish [37]. Such a stress response comprises of activation of the autonomic nervous system, as well as the hypothalamus-pituitary-interrenal (HPI) axis. About 25 years ago, it was not yet known how the neuro-endocrine system was interacting with the immune system of fish. The group of Płytycz (Table 1, Figure 10) and Chadzinska (Table 2, Figure 11) was one of the first working in this area. They studied the number and activity of peritoneal leucocytes after intraperitoneal injection of a sterile irritant (thioglycollate) with or without morphine [39,40]. Such an artificial inflammation can be used as a disease and stress model. Under these circumstances, activated macrophages will release cytokines leading to further inflammation as well as activation of the HPI axis and the nervous system. Morphine injection caused a reduction in peritoneal leucocytes in goldfish and salmon and a higher survival rate of bacteria in the head kidney of goldfish. 

These observations indicate that an exogenous opioid such as morphine had modulating effects on the immune response in fish. Further studies in salmon and mice have shown, that morphine-inhibited influx of leucocytes into the peritoneal cavity correlates with morphine-lowered level of plasma chemotactic factors both in fish and mice. The inhibitory effects of morphine on both the cell number immune organs and chemo-attractant level were completely reversed by naltrexone pretreatment, which implicated the involvement of opioid receptors [41]. In 2009, the existence of three classical opioid receptors (OR) in carp was shown by cloning and sequencing of the respected genes [42]. Both in brain and in immune organs constitutive expression of these OR genes was observed. During zymosan-induced peritonitis expression of OR genes in leucocytes was upregulated. Specific agonists of OR reduced leucocyte migratory properties. These data indicate an evolutionary conserved role for the opioid system in maintaining a dynamic equilibrium while coping with stress and/or infection.

Exciting observations were made when carp genomes were screened for sequences of the chemokine, CXCL8 [43]. Two different CXCL8 lineages were retrieved and for both cytokines indications for an early inflammatory function were found. Recombinant proteins of carp CXCL8 were produced and showed significant chemotactic activity for carp leucocytes. For a comprehensive review on neuro-endocrine immune interactions in fish see [44].

## 7. Genetic Aspects of Immunity

Several examples of genetic differences in disease resistance in fish have been described. One of the best examples of breeding for resistance to “infectious dropsy” in carp comes from Kirpichnikov *et al.* [46]. Healthy fish from three carp strains (Local, Ropsha and Ropsha × Ukranian) were selected for resistance to “dropsy”. The best results were obtained with the Ropsha × Ukranian cross after five generations of selection. In another study by Wiegertjes, Pilarczyk (Figure 5), and Van Muiswinkel it was shown that in a comparison of two inbred carp lines (W and R8) and their hybrid (W × R8) the W carp were most susceptible to a laboratory challenge with atypical *A. salmonicida*. Survival of the W carp in a winter field test was also much lower than the survival of the R8 or hybrid carp [47].

**Figure 12:**
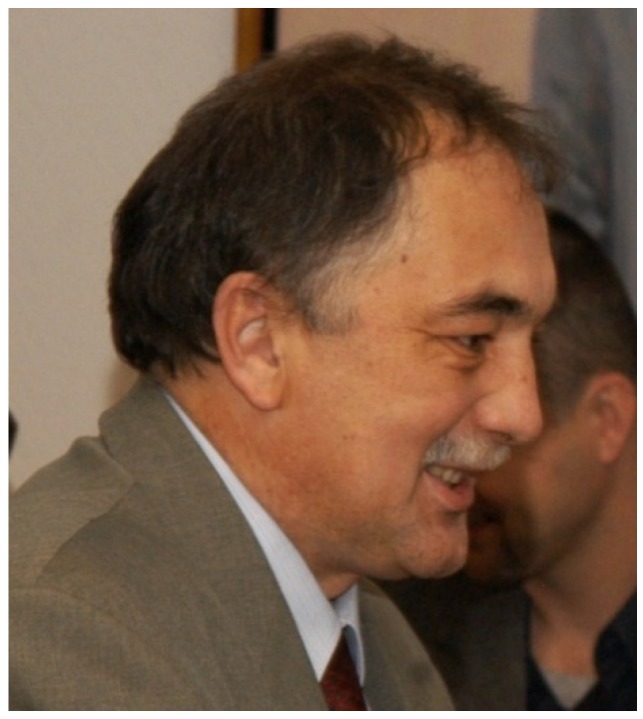
Ilgiz Irnazarow received his Ph.D. degree in Agricultural Sciences based upon a thesis entitled “Genetic markers of common carp” from the Institute of Genetics and Animal Breeding, Jastrzębiec (Poland) in 1994. In the period between 1989 and 1997 he started as production trainee at the Regional Centre for Agricultural Development, Bielsko-Biala (Poland) and became subsequently researcher at the Institute of Ichthyobiology and Aquaculture, Polish Academy of Sciences (PAS), Chybie (Poland). From 1997 until 1999 he joined the Faculty of Fisheries, Hokkaido University, Hakodate (Japen) as JSPS postdoctoral fellow. In 1999, he returned to the Institute of Ichthyobiology and Aquaculture (PAS) and became acting director of this institute in 2011. He can be regarded as a specialist in sustainable aquaculture, fish immunology, and diseases. He and his coworkers have published excellent work on the genetic aspects of immunity and disease resistance in fish.

The identification of genes involved in the regulation of defense mechanisms is important for our understanding of disease resistance. Studies in mammals have shown, that genes of the major histocompatibility complex (MHC) play an important role in the regulation of the immune response. Moreover, an association between certain MHC alleles and the susceptibility for specific diseases has been established in mammals [48]. In recent years, remarkable progress was made in the study of comparable genes in fish. However, in the teleost genome these MHC genes are organized in a very different manner compared with the case in mammals [49]. In fish, the major histocompatibility (MH) genes are not linked in a complex, but are unlinked. There are now examples showing that MH gene polymorphism in fish can be linked with disease resistance. In a study from the group of Irnazarow (Figure 12) and Pilarczyk (Figure 5) it was possible to link certain MH class II genotypes in carp with resistance to the ectoparasite, *Argulus japonicus*, or the sensitivity to the blood parasite, *Trypanoplasma borreli* [51]. In the same year, 2009, Rakus *et al.* [52] were investigating the role of MH class IIβ (*Cyca-DAB1-*like) genes in resistance of carp to cyprinid herpesvirus-3 (CyHV-3, also known as koi herpesvirus, KHV). By challenging six carp crosses with the herpes virus one *DAB-like* genotype was found associated with higher resistance while three other genotypes with lower resistance to the virus were found. From the same research group at Gołysz (PL), we also know that there are additional genetic polymorphisms which could play an important role, e.g., transferrin (Tf) polymorphism [53]. Most of the iron in serum is bound to Tf and at the same time Tf is an important growth factor for blood parasites, such as *T. borreli*. Using Tf genotyping in a series of carp lines, they were able to show that a homozygous Tf genotype (DD) was associated with low parasitemia. It is great to see that more and more genetic markers became available for breeding fish for resistance to diseases in the future. A recent and exciting finding is dealing with differences in the stress response in carp lines known to express different susceptibility to disease [54]. In the pathogen-resistant K carp line, there was a striking effect of stress on leukocyte composition and activity, even though no robust changes in gene expression of stress-involved factors were observed. In contrast, the most susceptible R3 carp line showed no spectacular changes in their immunological parameters with concurrent significant activation of the hypothalamus-pituitary-interrenal (HPI) axis. In other words: the highest stress response is found in fish with the highest susceptibility to disease.

## 8. International Collaboration

There is no doubt that the contacts between individual scientists from different countries play an important role in the progress of science. It is obvious, that this also holds true for scientists in Poland. Numerous papers have been published illustrating the effective co-operation with colleagues from abroad. In this review we will limit ourselves to a few successful examples:

(**a**) In the period between 1986 and 2003, Barbara Płytycz (Figure 10) and her coworkers published 26 papers together with Rolf Seljelid’s group at het department of Medical Biology, Arctic University of Norway, Tromsø (Norway). These studies cover a wide range of subjects from the field of Comparative Immunology, including fish, amphibians, and mammals. Special attention was payed to stress, inflammation, and the role of macrophages [39,41]. One of their reviews appeared in the high ranking journal “Immunology Today” [55].
(**b**) Another example comes from Andrzej Siwicki (Figure 8), who wrote 16 publications together with Doug Anderson, U.S. Fish & Wildlife Service, National Fish Health Research Laboratory, Leetown (WV, USA) in the years 1989–1996. These studies were dealing with important subjects such as diagnosis of bacterial and viral diseases [56], immunomodulation, and protection against infectious diseases by natural and synthetic products [57]. An amazing success was their publication from 1994 (with Gary Rumsey, U.S. Fish & Wildlife Service, Tunison Laboratory of Fish Nutrition, Cortland, NY, USA as co-author) on the use of immunostimulants in trout for protection against furunculosis [25]. This paper was cited > 460 times according to Google Scholar in 2015.
(**c**) Siwicki and coworkers also performed an impressive series of studies in collaboration with Marc Morand c.s., Laboratoire départemental d’analyses véterinaire, Conceil général du Jura, Lons-le-Saunier (FR). During the years 1996–2004 they published around 45 papers on subjects such as immunomodulation [58,59] and immunotoxicology [60]. The results of these studies could be used for several applications in aquaculture.
(**d**) Magdalena Chadzińska (Figure 11) has developed a very successful co-operation with Lidy Verburg-Van Kemenade, Cell Biology & Immunology Group, Wageningen University-WUR, Wageningen (NL) during the last 10 years. They and their staff have trained numerous MSc and PhD students and published more than 20 papers on subjects dealing with macrophages, inflammation, and neuro-immune interactions in fish [42,43,44,54,61].
(**e**) The Institute of Ichthyobiology & Aquaculture in Gołysz, Polish Academy of Sciences, Chybie (PL) (Project leaders: Andrzej Pilarczyk, Figure 5; Ilgiz Irnazarow, Figure 12) has been co-operating with several foreign groups supported by the EU Marie Curie Training Network PARITY “Integrated approach to the innate immune response to parasites in fish” (2002–2006) and NEMO “Training network on protective immune modulation in warm water fish by feeding glucans” (2008–2012). Important partners in these programs were: —Institute of Science and Technology in Medicine, School of Life Sciences, Keele University, Keele (UK) (Project leader Dave Hoole); —Cell Biology & Immunology Group, Wageningen University-WUR, Wageningen (NL) (Project leaders Geert Wiegertjes, Huub Savelkoul); —Fish Diseases Research Unit, University of Veterinary Medicine, Hannover (DE) (Project leader Dieter Steinhagen). This collaboration has produced more than 20 publications, showing that genetic factors (e.g., transferrin polymorphism, major histocompatibilty genes) play an important role in the disease resistance of carp [53,62]. Special attention was also payed to the role of acute phase proteins (CRP) in resistance to bacteria and viruses [63,64] and the stimulating effect of dietary glucans on the immune system of fish [65,66].

## 9. Conclusions

Polish researchers have played an important role in the development of fish immunology as a science during the last 100–120 years. They have been interested in the basic aspects of fish immunology (cells and molecules involved in immune responses, evolution of the immune system), but the practical application of their studies (e.g., vaccination, immunostimulation, breeding for disease resistance, effects of drugs, pollution, stress) have always been in their mind. This is not surprising in a country where aquaculture has been a traditional part of agriculture. Another factor for their success is certainly their ability to take part in several co-operative international research programs (see also 8 international collaboration). For the future it can be expected that young and promising researchers, *i.e.*, Adamek [52,64], Guz [22,23,29,30], Jurecka [51,53,54], Rakus [51,52,62] and many others will continue to produce interesting and prominent results. There is no doubt that our knowledge about the immune system of fish can be used in the future for the evaluation of the health status of fish under different conditions, disease prevention, and the promotion of fish welfare in aquaculture not only in Poland, but in the rest of the world as well.
